# CONTRIBUTION OF CHRONIC CONDITIONS TO MORTALITY: DIFFERENCES BY RACE AND ETHNICITY

**DOI:** 10.1093/geroni/igad104.2468

**Published:** 2023-12-21

**Authors:** Ana Quiñones, Miriam Elman, Gail McAvay, Brent Vander Wyk, Corey Nagel, Heather Allore

**Affiliations:** OHSU-PSU School of Public Health, Portland, Oregon, United States; OHSU-PSU School of Public Health, Portland, Oregon, United States; Yale University, New Haven, Connecticut, United States; Yale University, New Haven, Connecticut, United States; University of Arkansas for Medical Sciences, Little Rock, Arkansas, United States; Yale University, New Haven, Connecticut, United States

## Abstract

Health disparities exist in the timing, order, and accumulation of chronic somatic and neurodegenerative conditions, which are associated with increased health care utilization, expenditure, and mortality. We assessed the contribution of coexisting conditions to mortality among Hispanic, non-Hispanic (NH) White, and NH Black older adults in the United States to measure their burden. We used nationally-representative data from the Health and Retirement Study (HRS) from 1998-2016 (n=11,532). This analysis estimates the absolute additive contributions of seven somatic and two mental/neurodegenerative conditions using a longitudinal average attributable fraction derived from a survey-weighted multivariable discrete survival model adjusted for sociodemographic characteristics (sex, education, net worth, body-mass index, coupled, insured, and any of six ADLs lasting three or more months “because of physical, mental, emotional, or memory problems”). Conditions accounted for over 60% of mortality in each racial/ethnic group (64.0%, 62.2%, and 70.5% in Hispanic, NH White, and NH Black adults, respectively). Although we found that cardiovascular diseases (heart disease and hypertension) were the greatest contributor to morality in each racial/ethnic group, the impact of other conditions varied by race/ethnicity. Leading contributors to mortality were diabetes (12.0%) and dementia (7.2%) for Hispanic participants; cancer (13.0%) and lung disease (10.7%) for NH White participants; and diabetes (11.5%), lung disease (7.4%), and dementia (7.3%) in NH Black participants. This study provides insights into racial and ethnic differences in the contribution of chronic somatic and neurodegenerative conditions to mortality and may help target efforts for specific preventative care services to populations most impacted by them.

